# Microbiology of Middle Meatus in Chronic Rhinosinusitis

**DOI:** 10.1016/S1808-8694(15)30108-7

**Published:** 2015-10-19

**Authors:** Elisabeth Araujo, Celso Dall, Vladmir Cantarelli, Alexandre Pereira, Afonso Ravanello Mariante

**Affiliations:** a Otorhinolaryngologist. M.S. and PhD in Medicine. Professor of the Postgraduate program in Medicine of the Federal University of Rio Grande do Sul - UFRGS.; b Otorhinolaryngologist. PhD in Medicine, Head of the Department of Otorhinolaryngology and Head and Neck Surgery of the HCPA.; c PhD in Microbiology, Adjunct Professor - Feevale.; d MD. Resident Physician in General Surgery HCPA.; e Otorhinolaryngologist. M.S. student at the Postgraduate Program in Medicine UFRGS.

**Keywords:** meatus, microbiology, middle, rhinosinusitis

## Abstract

This was a prospective study which assessed endoscopically collected middle meatus secretions in patients with chronic rhinosinusitis (CRS) and compared those findings with microbiological data of healthy individuals.

**Methods:**

Middle meatus samples were collected from 134 CRS patients. In the laboratory, samples were Gram stained for microscopic examination with white blood cels (WBCs) count and also send for aerobic, anaerobic and fungal cultures. Fifty volunteers served as control.

**Results:**

In CRS patients a total of 220 microorganisms were isolated. The most frequent microorganisms were Staphylococcus aureus (31%), coagulase-negative Staphylococcus (CNS) (23%). Gram-negative or facultative microorganisms were isolated in 37% of the samples, anaerobes in 12% and fungi in 14%. Seventy four percent of the samples with positive cultures presented many or few WBC. In the control group, 76% of cultures were positive for aerobes and 12% for fungi. No anaerobes were isolated. There were rare or no WBC in the fifty samples. The most frequent microorganisms were CNS (40%), Staphylococcus aureus (18%).

**Conclusion:**

The microbiology of the middle meatus is similar in CRS patients and healthy individuals. Despite this, there was an important difference between the WBC count in these two groups, which helps to distinguish an infective from a saprophitic microorganism.

## INTRODUCTION

Characteristically speaking, patients with chronic rhinosinusitis are submitted to prolonged courses of antibiotics, and this favors the selection of resistant strains. This fact, together with patient non-compliance to treatment, the use of inefficient drugs, medication used in wrong doses, and the injudicious use of antibiotics have all contributed to a lower sensitivity to antibiotics by microorganisms.

Nasal endoscopy brought about a new horizon to nasal culture, since it allows for secretion harvesting directly from its drainage site, with minimum patient discomfort. Many studies have been carried out aiming at assessing the use of endoscopy in investigating the microbiology of rhinosinusitis, especially in chronic rhinosinusitis. However, interpreting the results from these studies is hampered because there is no standardization in the classification of the groups studied, the methodology used, sample harvesting site, and means used to sterilize the instruments used in the nasal mucosa. Moreover, it is difficult to carry out a microbiological assessment of the patients who are under use of antibiotics. Thus, the study of the bacterial flora from the middle meatus of healthy individuals is important in order to determine the micro-organisms that colonize the paranasal cavities, and to monitor their level of antibiotic resistance.

This study was carried out aiming at identifying the bacterial flora present in the middle meatus of patients with chronic rhinosinusitis and in healthy subjects. This was done by comparing the microbiology between the samples collected by nasal endoscopy and the samples harvested directly from the ipsilateral maxillary sinus, through a punction on the canine fossa and the microbiology of patients with chronic rhinosinusitis and the control group.

## METHODS

We assessed 134 patients with chronic rhinosinusitis retrospectively between March of 1999 and March of 2005, in Porto Alegre, Brazil.

Inclusion criteria were: signs and symptoms of rhinosinusitis lasting for at least 3 months, non-respondent to drug therapy with augmented amoxicillin and/or second generation cephalosporin for 4 weeks^1^; paranasal sinuses opacity seen at the CT Scan^2^; and the presence of middle meatus secretion seen at the nasal endoscopy^3^, ^4^, ^5^, ^6^. We excluded patients who had used antibiotics in the 21 days that preceded material collection and /or who presented septal deviation that impaired middle meatus visualization^7^, ^8^. CT scan was carried out 24-72h after sample collection^9^.

Among the patients included in this study, 16 patients who had ipsilateral maxillary sinus opacification were submitted to the collection of secretions in this sinus through a punction of the canine fossa.

In order to form the control group, we included 50 healthy individuals without prior history of acute or chronic rhinosinusitis, nasal allergies, cystic fibrosis, nasal polyps, cilliary diskinesia, AIDS or diabetes mellitus, and those who had not used antibiotics, steroids, antineoplastic drugs, nasal sprays or cocaine within 21 days prior to the study^10^, ^11^. We also excluded patients with septum deviation that impaired visualization of the middle meatus^8^.

Samples were collected through endoscopy (Storz, 4mm or 2.7mm, scopes with 30º or 0º angle). The mucosa was previously anesthetized with a cotton ball soaked in 4% neotutocaine left in the nasal cavity for 5 minutes^12^, ^13^.

Middle meatus secretions were collected from one side only, by means of a sterilized 2mm suction tip coupled to the Specimen Trap model 076-0490 (Sherwood Medical, St. Louis, EUA) collection container.

In 16 patients, secretions from the ipsilateral maxillary sinuses were collected by means of a trocar inserted through the canine fossa, after the placement of cotton balls soaked in 4% neotutocaine, using the same collecting mechanism^8^. The maxillary sinus was not flushed prior to secretion aspiration.

In the control group of individuals without rhinosinusitis, cotton balls soaked in 4% neotutocaine solution were introduced in the nasal cavity and, afterwards, the scope was used to push the nasal wing, thus reducing the possibilities of sample contamination by the nasal vestibule and the vibrissae. Middle meatus material collection was carried out with a swab directly placed in the middle meatus until its fibers became moist^10^, ^11^.

Samples were referred to the laboratory no later than 1 hour after harvesting, in a Stuart (Starplex Scientific, Ontario, Canada) transportation means for the rearing of aerobic microorganisms; and in thyoglycolate broth for anaerobic rearing.

In the laboratory, the bacterioscopic exam was carried out using the Gram dye. Leucocytes were determined by a semi quantitative technique, with classification in three groups: absence, rare and numerous^10^, ^14^, ^15^.

For aerobic culture, the material was spread in plates with McConkey agar (Difco, Detroit, USA or Becton Dickinson, Maryland, USA) and Tripticase Soy agar (Difco, Detroit, USA) enriched with 10% of sheep blood (blood agar) and incubated at 37oC for 24 hours. If no bacterial growth was seen, the mediums were re-incubated for 24 hours more before being deemed negative.

Anaerobe germs rearing was carried out in sheep blood agar, having as base the brucella blood agar (Difco, Detroit, USA) and the bacteroid bile esculin agar (BBE), with incubation for the most of 72 hours in anaerobiosis atmosphere provided by the following systems: Gaspak (Becton Dickinson, Maryland, USA), Anaerocult (Merck SA, Brazil) or Anaerobac (Probac, São Paulo, Brazil). After isolation and confirmation of its anaerobic nature, the microorganism was identified using the API system for anaerobes (Bio Merieux, France).

Mycology studies were carried out through direct material examination in slide and micro-slide; and material culture in Saboraud medium with or without chloramphenicol and cycloheximida (BBL). Incubation was carried out under 25°-35°C, and the cultures were observed for up to 20 days before being considered negative for fungi. Fungi and yeast identification was carried out through microscopic morphology and the use of a commercially available kit to identify yeasts (API system, Bio-Merieux, France), respectively.

Determining the minimum inhibitory concentration (MIC) and bacterial identification were carried out by automation (MicroScan, AutoScan-^4^) through isolating the bacteria in BHI (Brain Heart Infusion Broth), incubated at 35°-37°C for 6-12 hours and inoculated in panels appropriated for gram-positive or gram-negative germs.

Laboratorial procedures followed those advocated by the National Committee for Clinical Laboratory Standards (NCCLS), considering the bacterial species and the site of infection^16^. Data was examined focusing on the positive cultures and the identification of microorganisms. Middle meatus cultures were analyzed in isolation, and were also compared with the results obtained from the Gram study with those from the maxillary sinus and those from the control group. We used the Epi info 6.0 statistical package, and Yates-corrected chi-squared test to compare ratios, and Fisher’s exact test17. Acceptable error was of 5% (p < 0.05). Fischer’s exact test was used when the expected number for a certain trait was less than 5 patients^14^.

We calculated sensitivity, specificity and predictive values for middle meatus culture.

During the individual assessment of eligible patients, we requested them to sign an informed consent form. The volunteers were educated about the study and gave their verbal consent, and none of them were compensated. The project’s protocol was approved by the Ethics Committee.

## RESULTS

One or more samples were collected from all patients with chronic rhinosinusitis in the study. Thus, 168 samples were submitted to aerobe germs culture, 144 for anaerobes and 114 for mycology exam. Eight percent of the samples were sterile and 17% had mixed flora.

We cultivated a total of 220 microorganisms (Table 1): Staphylococcus aureus, the most frequently found aerobe, was present in 31% of the samples; coagulase-negative Staphylococcus was present in 23% and Streptococcus pneumoniae in 13%. Gram-negative or facultative microorganisms were identified in 37% of the samples. Anaerobe germs were isolated in 12% of the samples.

**Table 1:** Microorganisms isolated from the middle meatus of patients with chronic rhinosinusitis

# of Species isolated
Gram-positive aerobes
Staphylococcus aureus53
Streptococcus pneumoniae22
Staphylococcus epidermidis19
Streptococcus viridans6
Staphylococcus sp6
Streptococcus agalactiae3
Streptococcus intermedius3
Staphylococcus auriculars
Staphylococcus haemolyticus2
Staphylococcus lugdunensis2
Enterococcus avium1
Staphylococcus xylosus1
Streptococcus pyogenes1
Aerococcus viridans1
Enterococcus faecallis1
Enterococcus gallinarium1
Enterococcus avium1
Eikenela1
A. xilosidans1
Facultative Gram-negative aerobes
Pseudomonas aeruginosa10
Moraxella catarrhalis13
Haemophylus sp.11
Escherichia coli4
Enterobacter aerogenes3
Acinetobacter iwollfii2
Tatumella ptyseos2
Serratia fanticola2
Citrobacter freundii1
Enterobacter cloacae1
Enterobacter intermedium1
Klebsiella oxytoca1
Morganella morganii1
Proteus mirabilis1
Providencia alcalifantis1
Serratia marcescens1
Burkholderia cepacia1
Stenotrophomonas maltophilia1
Gram-positive Anaerobes
Peptostreptococcus sp.5
Peptococcus2
Actinomyces1
Gram-negative anaerobes
Bacteriodes fragiles6
Fusobacterium varium1
Prevotela bivians1
Prevotela oris1
Prevotela sp.1
Fungii
Candida sp.6
Aspergilus sp.2
Aspergillus niger1
Aspergillus fumigatus1
Eutipela1
Paecilomyces1
Alternaria1
Fusarium1
Penicillium 1
Schizofilum comuni1
Tricoderma viridans1

Total 220

Fungi were identified in the middle meatus samples from 14% of the patients with chronic rhinosinusitis. The comparative study results of direct and culture mycology are summarized on Table 2. Fungal rhinosinusitis was classified as allergic in four cases, and as fungal ball in three. In nine patients fungi were considered colonizing for having negative direct exam and because the patients did not have evidence of fungal rhinosinusitis. In one patient, hyphas and yeast were identified under light microscopy, however, culture was negative.

**Table 2:** Comparison between culture and direct mycology exams in middle meatus samples from patients with chronic rhinosinusitis

Fungi	Direct visualization	Culture
Candida sp.	---- + +	6
Aspergillus sp.	+ +	2
Aspergillus niger	-	1
Aspergillus fumigatus	+	1
Alternaria	-	1
Fusarium	-	1
Penicillium	-	1
Schizofilum comuni	+	1
Tricoderma viridans	+	1
Eutypela	-	1

### Leukocyte Count by the Gram Method

Analyzing leukocyte count by the Gram Method, 41% of the samples presented numerous leucocytes, 33% had some; 9% had extremely few; and 17% had no leucocytes. Among positive culture samples, 73% showed numerous or some leucocytes indicating infection. In the group with rare or no leucocytes at al, there were 41% of coagulase-negative Staphylococcus and 28% Staphylococcus aureus.

### Comparison between samples from the middle meatus and those from the maxillary sinus

We sampled cultures through the canine fossa in 16 patients, 18 from the maxillary sinuses and ipsilateral middle meatus. In our comparative study, 75% of the germs were the same in both cultures. In three aspirates, at least one of the germs was the same in both cultures (Table 3).

**Table 3:** Comparison among cultures of samples obtained from the middle meatus and the maxillary sinus.

Case #	Middle Meatus	Maxillary Sinus	Concordance
1	Streptococcus agalactie	Streptococus agalactie	+
2	Enterococcus gallinarium	Enterococcus gallinarium	+
3	Staphylococcus aureus Streptococcus pneumoniae	Scedosporium apiosperm	-
4	d - Actinobacter	Actinobacter	+
	e - Actinobacter	Actinobacter	+
5	d - Streptococcus viridans	Negativo	-
	e - Peptostreptococcus	Streptococcus pneumoniae	-
6	Pseudomonas aeruginosa	Pseudomonas aeruginosa	+
7	Streptococcus milleri Tricoderma sp.	Streptococcus milleri Tricoderma sp.	+
8	Staphylococcus aureus	Staphylococcus aureus Aspergillus	±
9	Staphylococcus aureus	Staphylococcus aureus	+
10	Staphylococcus aureus	Staphylococcus aureus	+
11	Negative	Negative	+
12	Pseudomonas aeruginosa Fusarium	Pseudomonas aeruginosa Pseudascherella boydii	±
13	Serratia marcescens	Serratia marcescens	+
14	Negative	Negative Pseudomonas aeruginosa	±
15	Staphylococcus sp (coagulase -)	Staphylococcus sp (coagulase -)	+
16	Staphylococcus haemolyticus	Staphylococcus haemolyticus	+

### Control Group

Aerobic microorganisms and fungi were identified in 76 and 12% of healthy volunteers, respectively. In terms of the semi-quantitative leukocyte count, 14% of the samples presented rare leucocytes and 86% had none. Coagulase-negative Staphylococcus was the most common microorganism found, present in 40% of the individuals. Staphylococcus aureus, Corynebacterium sp. and Corynebacterium pseudodiphteriticum were identified in 18, 12 and 6% of the samples. No anaerobe germ was isolated.

Comparison among control group microorganisms and those found in patients with chronic rhinosinusitis

In the comparative study of leukocyte count by the Gram method, the control group presented statistically significant difference, with 86% absence and 17% in patients with chronic rhinosinusitis (p < 0.0001).

Coagulase-negative Staphylococcus was statistically more frequent in the control group when compared to patients with chronic rhinosinusitis (p < 0.009), while the Gram-negative germs were the most frequent in patients with chronic rhinosinusitis (Table 4).

**Table 4:** Comparison between the microorganisms isolated from the control group and the group of patients with chronic rhinosinusitis.

Microorganisms	Chronic rhinosinusitis (n = 134) %	Control (n = 50) %	p
Aerobes	81	76	0.3894**NS
Staphylococcus coagulase-negative	23	40	0.0096**
Staphylococcus aureus	31	18	0.0325**
Streptococcus pneumoniae	13	0	0.0002*
Gram-negative	37	18	0.0026**
Anaerobes	12	0	0.0003*
Fungi	14	12	0.6741**NS

p = Pearson’s p-values for the chi-squared test

NS = Statistically non-significant difference

* Fisher’s exact chi-squared test

** Yates’s chi-squared correction

## DISCUSSION

Traditionally, the direct punction with aspiration method and/or biopsy of the maxillary sinus mucosa has been employed as the gold standard in determining the microorganisms involved in rhinosinusitis. However, such technique bears severe limitations, since it does not provide data on the microbiology of the ethmoid, frontal and sphenoid sinuses. On the other hand, some less invasive techniques to obtain culture material from the paranasal sinuses, such as nasal or nasopharynx swab sweeping are not reliable because of the high rate of contamination by colonizing microorganisms^5^, ^18^, ^19^, ^20^. Nasal endoscopy allows for the identification of structural alterations that predispose a patient to chronic rhinosinusitis and the visualization of the paranasal sinuses drainage areas. This procedure may be easily carried out under local anesthesia in the office, with minimum discomfort^21^. Moreover, the validation of sample cultures collected by means of middle meatus endoscopy will represent a major step in handling recurrent rhinosinusal infections.

Percentage growth of Staphylococcus aureus in cultures has varied from 1% to 29% with the Caldwell-Luc^22^, ^23^, ^24^, ^25^ technique; and between 15% and 52% in anterior rhinoscopy^24^, ^26^, ^27^. In middle meatus cultures endoscopically sampled from the middle meatus, Bolger et al.^28^ found12% of Staphylococcus aureus, Hsu et al.^29^ and Klossek et al.^6^ cultivated 19% and Nadel et al.^14^, 23%. In our study, Staphylococcus aureus grew in 31% of the cultures and, in the control group made up of healthy individuals, it was cultivated in 18% of the time, however leukocyte count and/or the semi-quantitative culture was absent or rare.

Coagulase-negative Staphylococcus isolated in nasal secretion cultures are usually considered as a contaminant, although in other organs its role as a pathogen is well documented ^29^. Nadel et al.^14^ found coagulase-negative Staphylococcus in 35% of the middle meatus cultures. Klossek et al.^6^ found in patients with chronic rhinosinusitis, 24% of coagulase-negative Staphylococcus and 46% in the control group, also with a statistically significant difference (p < 0.001). These findings reinforce the concept that coagulase-negative Staphylococcus are predominantly saprophytes; however, when found with numerous leucocytes and/or massive growth, may represent a true infection.

Gram-negative have an important role in chronic rhinosinusitis, especially in those cases that resist conventional treatment. Bolger et al.^28^, with the endoscopic culture technique, identified 31% of Gram-negatives, and they were the prevailing microorganisms. Authors such as Hsu et al.^29^, Nadel et al.^14^ and Gold and Tami^5^ reported rates between 26% and 32%, matching those found in the present study.

Enterobacteriaceae form an extremely variable group of facultative Gram-negative germs. In Chronic rhinosinusitis, they are associated with the release of citotoxic and proteolythic enzymes. These microorganisms are frequently isolated from hospital-acquired rhinosinusitis^28^. In the group of patients with community-acquired infections that we assessed, they were present in 8.5% of the aerobic cultures, and such finding was comparable to the 9% found by Nadel et al.^14^ and the 9% by Klossek et al.^6^.

The frequency of anaerobes in chronic rhinosinusitis microbiology studies vary between 0% and 100%. Brook et al.^30^ and Erkan et al.^8^, analyzing aspirates from maxillary sinuses, found anaerobes in 82% and 88% of the cases, respectively. In middle meatus cultures, anaerobic prevalence in literature papers is lower and is closer to the 12% we found. Klossek et al.^6^ reported 25%, Busaba et al.^31^ 13%, Muntz et al.^32^ 6%, Vogan et al.^9^ 6% and Rontal et al.^33^ 6%. Other authors^4^, ^5^ did not make anaerobe cultures from middle meatus samples. These conflicting results may be related to the differences in culture site and the techniques used.

Fungi were cultivated in 14% of the patients with chronic rhinosinusitis in this series, although only 6% were characterized as fungal rhinosinusitis. In the other patients, fungi were considered colonizing, since they were not seen under direct examination. Schell^34^ considers that positive culture for fungi related to negative microscopic exam may pose as a problem and should be analyzed case by case.

The sensitivity of cultures from secretions aspirated from the middle meatus by means of nasal endoscopy may be assessed by comparing it to the one obtained from maxillary punction. In the present investigation, in 16 patients we found 73% of the same microorganisms in both cultures. Similar results were published by Orobello et al.^13^, in children with 83% of concordance between middle meatus and maxillary antrum endoscopic cultures. Gold and Tami^5^ compared the samples from the middle meatus and the maxillary sinus of 18 patients with chronic rhinosinusitis and found a positive concordance of 85%. Klossek et al.^6^ concluded that the accuracy of endonasal endoscopic collection is close to 80%. These studies suggest that the endoscopic culture from the middle meatus is a feasible alternative to antral punction, since it is effective in identifying pathogens and is a non-invasive diagnostic method towards rhinosinusitis etiology.

As to the presence of fungi in the control group of the present study, they were cultivated in 12% of the individuals. However, in none of the direct examinations fungi were found, and they were all considered colonizing. Ponikau et al.^35^ isolated fungi by PCR in 100% of healthy individuals, identifying many species of Aspergillus, known contaminant or causal agent of allergic fungal rhinosinusitis. In the comparative study between the control group and the group with chronic rhinosinusitis, there was no statistically significant difference between the percentage of fungi isolated, finding which was similar to the one reported by Ponikau et al.^35^. Fergunson^36^ reports that fungi may be isolated from the nasal cavities of all individuals, however, these results must not be overestimated. Positive culture in completely asymptomatic individuals must be related to the clinical situation.

Despite successful results from the endoscopic surgery, about 3% to 20% of failure may happen, depending on follow up time and the type of patients analyzed. Surgical failure may manifest itself by symptoms persistence, recurrent infections and the need for revisional surgery. A considerable number of patients present, after endoscopic surgery, purulent secretion despite sinus ostium patency. Pseudomonas aeruginosa and Gram-negatives were identified as the most frequent germs in prior studies.^4^, ^14^, ^37^.

## CONCLUSION

Sample cultures from the middle meatus may be easily carried out, in an outpatient basis, in patients with rhinosinusitis and healthy individuals. This study shows that endoscopic guided paranasal culture sampling is well correlated with cultures from secretions collected directly from the maxillary sinus and may be used in selecting the antimicrobial medication.
